# Schottky Diode Leakage Current Fluctuations: Electrostatically Induced Flexoelectricity in Silicon

**DOI:** 10.1002/advs.202403524

**Published:** 2024-08-09

**Authors:** Carlos Hurtado, Melanie MacGregor, Kai Chen, Simone Ciampi

**Affiliations:** ^1^ School of Molecular and Life Sciences Curtin University Bentley Western Australia 6102 Australia; ^2^ Flinders Institute for Nanoscale Science and Technology Flinders University Bedford Park South Australia 5042 Australia; ^3^ School of Molecular Sciences The University of Western Australia Crawley Western Australia 6009 Australia

**Keywords:** flexoelectricity, Schottky diodes, silicon, surface chemistry, triboelectricity

## Abstract

Nearly four decades have passed since IBM scientists pioneered atomic force microscopy (AFM) by merging the principles of a scanning tunneling microscope with the features of a stylus profilometer. Today, electrical AFM modes are an indispensable asset within the semiconductor and nanotechnology industries, enabling the characterization and manipulation of electrical properties at the nanoscale. However, electrical AFM measurements suffer from reproducibility issues caused, for example, by surface contaminations, Joule heating, and hard‐to‐minimize tip drift and tilt. Using as experimental system nanoscale Schottky diodes assembled on oxide‐free silicon crystals of precisely defined surface chemistry, it is revealed that voltage‐dependent adhesion forces lead to significant rotation of the AFM platinum tip. The electrostatics‐driven tip rotation causes a strain gradient on the silicon surface, which induces a flexoelectric reverse bias term. This directional flexoelectric internal‐bias term adds to the external (instrumental) bias, causing both an increased diode leakage as well as a shift of the diode knee voltage to larger forward biases. These findings will aid the design and characterization of silicon‐based devices, especially those that are deliberately operated under large strain or shear, such as in emerging energy harvesting technologies including Schottky‐based triboelectric nanogenerators (TENGs).

## Graphical abstract

Electrostatic attractive forces between a Pt atomic force microscopy (AFM) tip and an oxide‐free silicon surface lead to a strain gradient in the semiconductor, which results in a flexoelectric junction voltage. Flexoelectricity increases diode leakage and shifts the Schottky junction knee voltage away from the origin, accounting for parasitic effects observed in nanoscale electrical measurements and guiding the design of triboelectric nanogenerators.

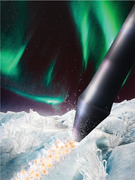

## 1 Introduction

Metal–semiconductor rectifying junctions—Schottky diodes—are a building block of many digital circuits and technologies.^[^ ^1^, ^2^ ^]^ While traditionally associated with certain data storage technologies and powering circuitry,^[^ ^3^ ^]^ Schottky diodes have also recently entered the field of energy generation.^[^ ^4^, ^5^ ^]^ In a nanoscale metal–semiconductor Schottky diode, relative motion of the two materials leads to a measurable direct current (DC) output, even under zero external bias.^[^ ^6^, ^7^ ^]^ Sliding diodes represent a significant category within the technology of triboelectric nanogenerators (TENGs).^[^ ^8^, ^9^, ^10^, ^11^ ^]^ The process through which a DC‐TENG based on a Schottky diode transforms mechanical energy into electrical energy is unclear, with the specific roles of friction,^[^ ^12^ ^]^ normal pressure,^[^ ^13^ ^]^ shear and strain,^[^ ^14^ ^]^ and their interactions, still being unclear. Specifically, what has recently emerged, is that surface polarization in response to bending—flexoelectricity— is not just limited to insulators, but occur also in semiconductors.^[^ ^15^, ^16^ ^]^ For instance, in 2016 Catalan and co‐workers demonstrated that in wide‐bandgap semiconductor oxides, flexoelectricity is augmented, rather than diminished, by increasing the material conductivity through doping.^[^ ^17^ ^]^

The DC output of a sliding Schottky diode increases as the junction contact area decreases,^[^ ^18^, ^19^ ^]^ hence nanoscale measurements, especially through atomic force microscopy (AFM), are ideally suited to study TENGs mechanisms and performances. The suitability of AFM‐based measurements in TENG research is strengthened by the possibility of simultaneously probing, with high spatiotemporal resolution, DC outputs, mechanical and electronic aspects of the junction, such as normal force, friction, and adhesion, as well as detecting the presence or absence of electronic defects.^[^ ^20^, ^21^, ^22^ ^]^ However, AFM‐based measurements, and not just in the context of TENGs but also within the broader realm of nanoscale electrical measurements, are often hindered by experimental factors that are hard to control or account for, such as tip‐tilting and bias‐dependent electrostatic interactions between probe and substrate.^[^ ^23^, ^24^ ^]^

Here we report experimental evidence of electrostatic forces between a conductive AFM tip and a silicon surface leading to mechanical instability of a nanoscale Schottky diode, and reveal that these forces account for a directional (relative to the diode's internal field) surface polarization. Regardless of the silicon doping type, the mechanically induced voltage term bears the direction of a reverse bias: it augments the external (operator‐defined) reverse bias and diminishes the external forward bias. We show how to detect, account for, and minimize (or augment) this parasitic polarization.

## 2 Results and Discussion

Asymmetric attractive forces between a semiconductor and metal‐like AFM tips have been reported before.^[^ ^25^, ^26^ ^]^ However, residual electrostatic effects—residual in the sense that they persist even upon the flow of an electric current—are poorly discussed in the literature.^[^ ^27^, ^28^ ^]^ A Schottky junction, just like any junction, has a certain ability to let charge through and to store charge.^[^ ^29^, ^30^ ^]^ Electronic drag is likely to have a negligible mechanical effect on the adhesion of a biased junction,^[^ ^25^ ^]^ so, if any, residual attractive forces are most likely capacitive in origin.

**Scheme 1:**
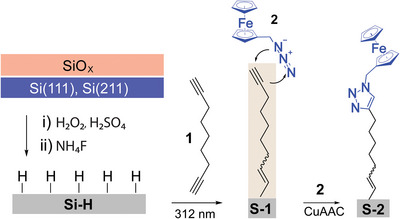
Schematic representation of the wet chemistry process for the passivation and functionalization of an oxide‐free Si(211) crystal. The removal of the native SiO*_x_* layer, followed by the UV‐assisted hydrosilylation of 1,8‐nonadiyne (**1**) on hydrogen‐terminated (Si─H) Si(211) and Si(111) yields a passivating (against anodic decomposition) but yet electrically conductive alkyne‐terminated monolayer (**S‐** **1**). The monolayer is further functionalized through a Cu‐catalyzed alkyne–azide cycloaddition (CuAAC) reaction to yield a ferrocene‐terminated monolayer (**S‐** **2**).

Since the capacitance of a Schottky diode depends on the external bias, our first objective was therefore to explore the magnitude and directionality of adhesion forces in diodes that were deliberately biased into either accumulation or depletion. The main experimental platform is shown in **Scheme** **1**, which depicts the chemical strategy used to passivate oxide‐free silicon wafers. In brief, silicon surfaces were first etched to yield a hydrogen‐terminated surface (Si─H), which has a low density of electrical defects but is unfortunately prone to oxidation (Figures S1 and S2, Supporting Information).^[^ ^31^ ^]^ The chemical reactivity of the Si─H surface can however be turned into a technical advantage, as it can be chemically passivated through surface hydrosilylation reactions with 1‐alkenes^[^ ^32^ ^]^ and 1‐alkynes^[^ ^33^ ^]^ monolayer‐forming molecules.^[^ ^34^, ^35^, ^36^ ^]^ The hydrosilylation of an α,ω‐dialkyne molecule (1,8‐nonadiyne, **1**, Scheme 1) on Si─H was used to prepare **S** **‐** **1** surfaces,^[^ ^34^ ^]^ where the acetylene moiety at the **S‐** **1** monolayer distal end also opens up the possibility of further chemical derivatization of the semiconductor surface. This option is here used to covalently tether ferrocene‐terminated monolayers (**S** **‐** **2**).^[^ ^37^ ^]^ The presence of a metallocene in the **S**‐**2** monolayer allows access to electrochemical measurements, which do not involve diffusive reactants and are therefore an unambiguous analytical tool to assess the outcome of the silicon passivation procedure. Electrochemical measurements done on **S‐** **2** samples also served to demonstrate that the chemical passivation of the Si(211) oxide‐free crystal does not preclude the flow of DC across the interface (Figure S3, Supporting Information).^[^ ^38^, ^39^ ^]^ The electrochemically determined monolayer surface coverage for **S‐** **2** samples is ≈2.2 × 10^−10^ mol cm^−2^, which is lower than the expected theoretical value for a closely packed ferrocene monolayer (4.5 × 10^−10^ mol cm^−2^)^[^ ^40^ ^]^ but in line with literature data for two‐step surface derivatization procedures.^[^ ^41^, ^42^, ^43^ ^]^ The full width at half‐maximum (fwhm) of cyclic voltammetry traces was ≈90 mV. This fwhm value indicates negligible interactions between the adsorbate molecules.^[^ ^44^, ^45^ ^]^ What is also evident in the voltammograms shown in Figure S3 (Supporting Information) is a peak “inversion”: the anodic wave peaks at more cathodic voltages than the cathodic wave. This inversion is indicative of electrostatic interactions between the silicon space charge region and the molecular charges of the adsorbate.^[^ ^42^ ^]^

**Figure 1:**
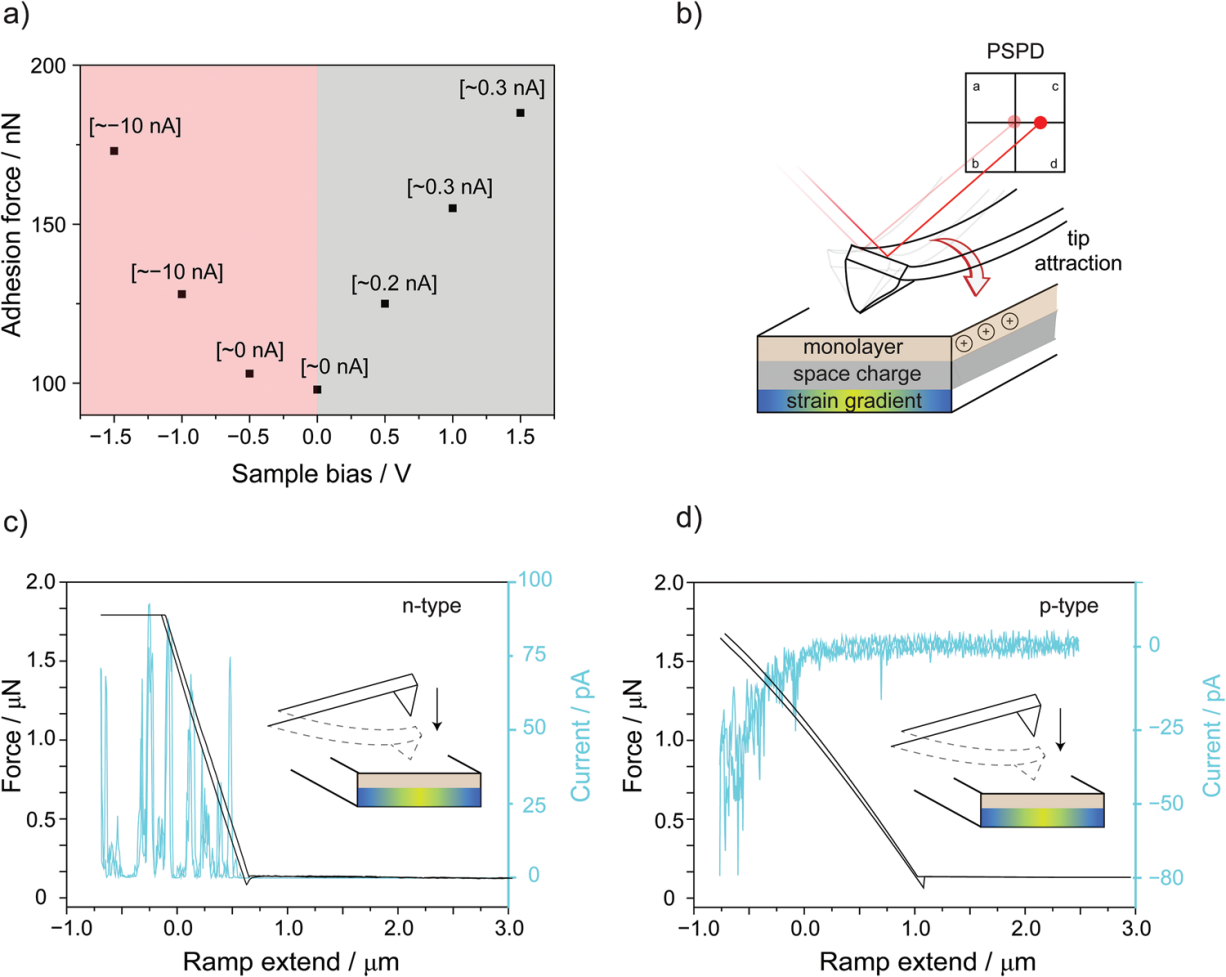
a) Tip‐sample (Pt AFM tip‐**S** **‐1** grafted on n‐type Si(211)) adhesion force data as a function of the external semiconductor bias (−1.5 to +1.5 V). Shaded areas delimit the forward (red) and reverse bias (gray) regimes. Labels next to each adhesion force–sample bias data point are the corresponding current values. b) Schematics depiction of a geometrically imperfect tip tilting laterally in response to a normal attractive force. The near‐surface positive charges of space‐charge ionized phosphorous dopants are indicated as plus symbols, and the blue/green color gradient indicates the strain gradient. c,d) High‐speed current capture data was acquired during AFM force–distance (*F*–*d*) measurements done on Si(211) **S‐** **1** monolayers prepared on either c) n‐type, or d) p‐type samples. The cyan traces represent the current flowing across the Schottky junction, under zero external bias, recorded as the platinum AFM tip is pressed down onto the silicon surface (*F*–*d* data, black traces). The instrument routing is such that a current of the positive sign indicates a flow of electrons from tip to sample.

After having validated the good quality of the semiconductor chemical derivatization, we then turned our attention to “dry” junctions. Data presented in **Figure** **1**a show changes in the adhesion force between the silicon surface and a platinum AFM tip as a function of the Schottky junction external bias. The junction's adhesion force was estimated by measuring the pull‐off force in AFM force–distance (*F*–*d*) experiments (Figure S4, Supporting Information), over a range of sample's bias voltages (from −1.5  to +1.5 V) covering from silicon accumulation to silicon depletion. The adhesion force displayed a quadratic voltage‐dependent characteristic, indicative of a capacitive behavior.^[^ ^23^, ^46^ ^]^ However, this bias‐dependent tip–sample electrostatic interaction does not follow a symmetric rise around the zero bias: for a given (absolute) value of voltage bias, adhesion forces are generally larger than when the diode is under reverse bias (grey region, Figure 1a) rather than under accumulation (red region, Figure 1a).

The original assumption of metal–silicon attractive forces being capacitive in nature is therefore in poor agreement with the data shown in Figure 1a, since the experimentally determined adhesion is greater when the junction is depleted, that is, when the interface capacitance drops and its resistance increases due to the silicon space‐charge region increasing in thickness.^[^ ^47^ ^]^ The observed tip–silicon attraction is greater when the Si(211) near‐surface region contains a population of unbalanced, immobile ionized phosphorous dopant atoms and opposes the flow of current^[^ ^48^ ^]^ (Figure 1a). If the thickness of this charged region is minimized by means of increasing the silicon doping level, the magnitude of the metal–silicon attraction also drops (Figure S5, Supporting Information).

One of the possible expected consequences of this bias‐dependent tip‐surface adhesion force—a lateral tip‐tilt due to the likelihood of an asymmetric tip shape—is schematically depicted in Figure 1b. Such tilting of the AFM tip may be detected by the instrument's position‐sensitive photodiode (PSPD) in the form of a change in the tracking laser deflection (vide infra). However, we first turned our attention to another plausible consequence of a mechanical movement caused by the observed tip‐substrate attraction. It is not unlikely that an increase in the normal force would cause a local strain gradient in the silicon substrate. For many materials, the consequence of a strain gradient (bending) is the generation of flexoelectricity. The first report on flexoelectricity is likely to be a 1910 paper by Walter Jamieson, who observed for celluloid sheets a curvature‐dependent surface charging.^[^ ^49^ ^]^ Flexoelectricity has been since then verified or anticipated for several materials^[^ ^14^, ^50^, ^51^, ^52^, ^53^, ^54^, ^55^ ^]^ including semiconductors.^[^ ^15^, ^16^, ^17^, ^56^ ^]^ For instance, Wu et al.^[^ ^53^ ^]^ have explored the modulation of device performance through flexoelectric effects in MoS_2_‐based metal–semiconductor field‐effect transistors (MESFETs), and Sun et al.^[^ ^52^ ^]^ have developed new methods to measure flexoelectric coefficients of semiconductors. But while a flexoelectric effect is likely to occur in strained silicon,^[^ ^16^, ^57^ ^]^ unfortunately the flexoelectric coefficient of silicon remains to be accurately determined. The reason for the lack of data on the magnitude and sign of the silicon flexoelectric coefficient is that a macroscopic strain–voltage measurement of such a brittle and hard material is hindered by the rapid formation of cracks.^[^ ^58^, ^59^ ^]^ However, as shown recently for n‐type SrTiO_3_, a strain gradient may alter local band bending, and since the flexoelectric surface polarization can increase (or decrease) the height and width of the interface Schottky barrier, strain gradients will likely affect charge transport characteristics (Figure S6, Supporting Information).^[^ ^60^ ^]^

To gain quantitative insights relevant to flexoelectricity in silicon we conducted *F*–*d* experiments while rapidly sampling the zero‐bias current. High‐speed force–current data (ramp speed 300 nm s^−1^) shown in Figure 1c and in Figure S7 (Supporting Information) show that, under no external bias, pressing down a platinum tip onto a Si(211) **S**‐**1** surface leads to a current response that bears the sign of the reverse current. It is not unlikely that pressure from the AFM tip (≈5 GPa) is causing a nonuniform strain near the tip‐surface contact region^[^ ^61^ ^]^ and that surface polarization due to the strain gradient (flexoelectricity) is the cause of the current flow. We note that the large zero‐bias current shown in Figure 1c is not an experimental artifact arising from the coupling of an increase in contact area (i.e., decrease in resistance) with a residual instrument “burden” voltage. As shown in Figure 1d, control force–current experiments performed on p‐type silicon revealed that the effect is directional: the direction of the putative flexovoltage changes by switching from n‐ to p‐substrate. These controls prove that the pressure‐related zero‐bias current is not caused by residual instrument burden voltage coupled to a drop in electrical resistance during the *F*–*d* scan. It is also unlikely for this current to have a thermal origin, such as a Seebeck voltage in response to a temperature gradient. The silicon Seebeck coefficient is ≈0.4 mV K^−1^, and to thermally generate currents such as those obtained in the *F*–*d* experiments of Figure 1 would require a junction potential of ≈1.7 V, which would in turn arise from a temperature difference as large as ≈3000 K.^[^ ^62^ ^]^

How the zero‐bias current output (Figure 1c,d) tracks the AFM tip force setpoint warrants a further discussion. Especially evident for junctions made on n‐type substrates, but to a lesser degree also for the p‐type systems, the flexoelectric current exhibits spike‐like features. There are two possible explanations for these current spikes. The first is an intrinsic technical limitation: the contact force is inferred from the vertical position of the cantilever rather than from the actual position of the platinum tip contacting the silicon substrate. Consequently, mechanical deformations, shear, and irregularities or abrupt changes in the nanoscopic motion of actual contact cannot be ruled out. Notably, the observation that current spikes are much less frequent in the p‐type system may also suggest an additional, although somewhat speculative, explanation, which is that in n‐type silicon the flexoelectric coupling is not linear with strain gradient.^[^ ^63^ ^]^

Having firstly demonstrated the presence of bias‐dependent attractive forces (Figure 1a) and then of an “internal” reverse bias term due to mechanical forces (Figure 1c,d), we went back to the hypothesis schematically depicted in Figure 1b: the possibility of an electrostatically driven lateral tip tilting during a static (i.e., not the typical mapping^[^ ^6^, ^12^ ^]^) AFM electrical measurement.

**Figure 2:**
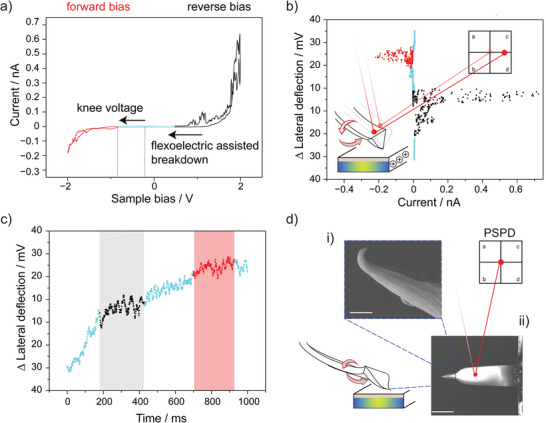
(a) Current–voltage (*I–V*) curves acquired at Pt AFM tip‐n‐type Si(211) **S**‐**1** junctions under an applied normal load of 100 nN at a voltage sweep rate of 8 V s^−1^. The expected asymmetric diode *I–V* trace is “inverted,” with the reverse bias current (black lines) comparable to, or greater than, the forward current. b) Lateral deflection–current plot revealing a substantial tip‐tilting during the *I–V* experiment shown in (a). The inset depicts the detection through the PSPD signal of the lateral tilt of the tip. The near‐surface positive charges of ionized phosphorous dopants in the space‐charge are indicated as plus symbols, and the color gradient indicates qualitatively the silicon strain gradient. c) Plot of the lateral deflection data shown in (b) as a function of time during the voltage ramp. Shaded areas indicate a “flatter” sections within the deflection curve which also correspond to maximum values of forward (gray) or reverse (red) current. d) SEM images of the Pt AFM tip obtained after the AFM measurements. Scale bars are 10 µm (i) and 100 µm (ii).

With this in mind we performed current–voltage (*I*–*V*) measurements while monitoring the tip lateral deflection channel. We found that poorly performing Schottky junctions, that is, “leaky” diodes with a poor rectification ratio (RR at 1 V below ≈0.3, **Figure** **2**a), were generally mechanically unstable as indicated by data on the tip lateral deflection (Figure 2b). A large diode's leakage, which we argue is the result of a flexoelectric voltage pushing electrons from the tip to the n‐type Si(211), was generally coupled to the presence of two discrete populations of tip‐tilt angles. As the external bias is ramped between the forward vertex (−2 V, sample bias) to the reverse bias vertex (+2 V), the tip tilts laterally until it reaches a deflection plateau (Figure 2b,c; tilting plateaus in red and black symbols) when a large forward or backward current effectively nullifies the electrostatic tip–semiconductor interaction. Mechanical movements of the tip are greater in between, i.e., when the flow of current is minimal (e.g., cyan symbols in Figure 2b). As mentioned earlier, the reason why a normal force causes a lateral, rather than normal, deflection is rooted in the imperfect geometry of the AFM tip, as revealed by representative scanning electron microscopy (SEM) images in Figure 2d. SEM data of the tip also help answering another important question: the cause of a delay between the end of the tip tilting event (as measured by the deflection channel) and the onset of the putative flexovoltage (as measured by the onset of an unexpected large leakage current). In other words, although a large reverse current is flowing—larger than expected based on the externally applied bias—while the tip‐tilt is “locked” in an apparent plateau, it is not unlikely that the end of the AFM tip (Figure 2d, inset‐i), which is in contact with the sample and located beyond the cantilever region probed by the AFM PSPD (Figure 2d, inset‐ii), is still rotating or sliding. We suggest that it is this “delayed” movement the cause of the flexoelectric surface polarization, which as shown by the current data acquired during *F*–*d* curves (Figure 1c) has the direction of a reverse bias. We note that this delayed movement is not affected by surface roughness. Control experiments done on Si(111) crystals, which are even smoother than the Si(211) substrates (rms roughness 0.3 nm vs 3 nm, Figure S8, Supporting Information) revealed similar tip lateral deflections and current outputs for the two platforms regardless of surface roughness (Figure S9, Supporting Information). Experiments with softer tips of the same radius (Figure S10, Supporting Information) showed larger zero‐bias currents, tentatively associated with an increased probability of the tip tilting in response to the electrostatic tip‐silicon attraction.

Regarding the nature of the electrical breakdown involved (i.e., the flexoelectric current has a direction of a leakage current), we attempted to elucidate whether the main mechanism is an avalanche or a Zener breakdown. For nominally static junctions biased externally (*I–V* curves), the leakage magnitude decreased with increasing temperature (Figure S11, Supporting Information) and for zero‐bias *F*–*d* curves we observed an enhanced flexoelectric response as the silicon space‐charge layer becomes thinner (i.e., higher dopant concentration) (Figure S12, Supporting Information). The effect of temperature and doping points towards a Zener rather than avalanche breakdown, where quantum tunnelling decreases as the depletion region widens.^[^ ^64^ ^]^ We also investigated the effect of changes to the normal load on the charge transport characteristics. *I–V* data in Figure S13 (Supporting Information) show that increasing the load increased both the leakage and the forward current: beyond a certain pressure threshold, the effect of an increased contact area overwrites any flexoelectric effect. Figures S14 and S15 (Supporting Information) show the C‐AFM maps at either the nullifying voltage (i.e., the external voltage required to bring the leakage current to noise level) or at zero‐bias, respectively. The open circuit voltage (*V*_OC_) and short‐circuit current (*I*_SC_) extracted from these maps scale with the applied load, ranging from 0.25 to 0.42 V, and from 0.2 to 1.0 nA (Table S1, Supporting Information).

**Figure 3:**
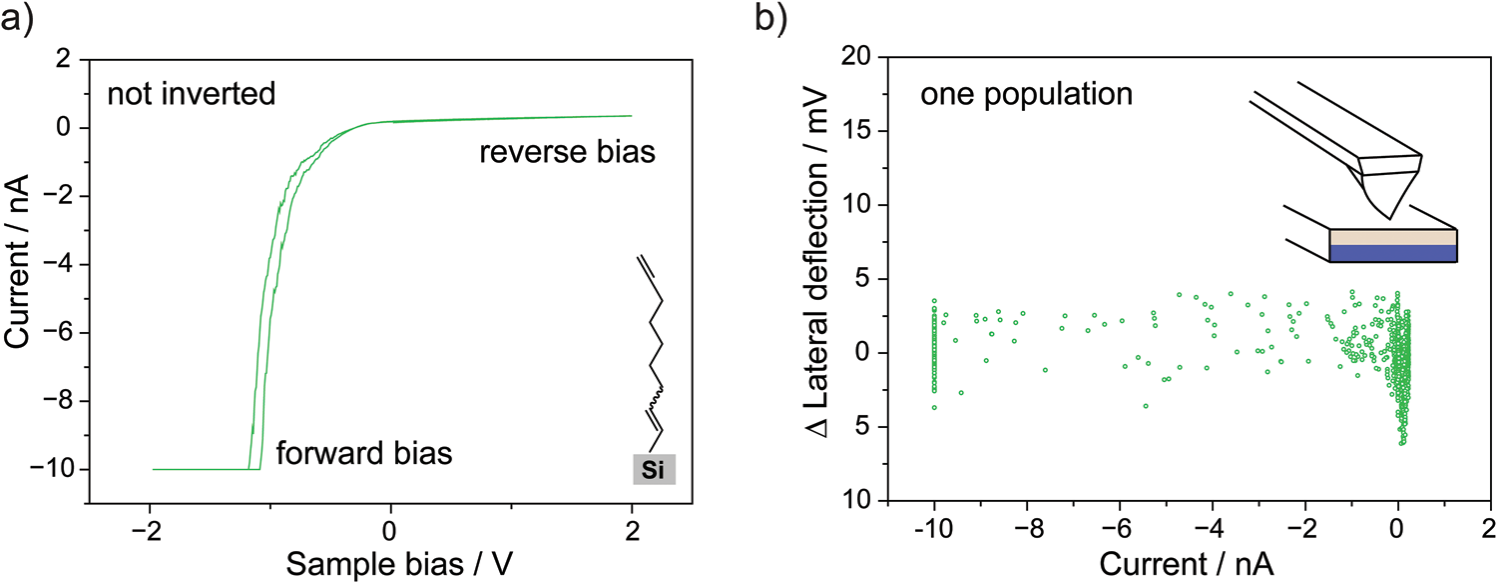
a) Representative *I–V* spectroscopy data acquired at a Pt AFM tip‐n‐type Si(211) **S**‐**1** junction under an applied normal load of 100 nN (voltage sweep rate of 8 V s^−1^). b) Lateral deflection–current plot showing negligible lateral tilting during the bias ramping. The inset schematically depicts the Pt AFM tip in static contact with the silicon surface when no tip tilting is observed.

To reinforce the role of electrostatics in causing mechanical instability (hence flexoelectricity) of the Schottky junctions, we conducted *I–V* measurements at a much lower scan rate (0.01 Hz), hence allowing additional time for the putative surface charges to dissipate. As shown in Figure S16 (Supporting Information), *I–V* inversion was rarely observed for slowly ramped junctions: only ≈1% of the *I–V* curves showed a measurable leakage current. Importantly, a normal diode behavior (RR is well above 1000) was also observed for nanoscale junctions that despite being ramped at a faster voltage sweep rate were mechanically stable (**Figure** **3**), as well as for macroscopic Schottky junctions under negligible normal force (Figure S17, Supporting Information). Also worth noting is the shift in knee voltage.^[^ ^65^ ^]^ The knee voltage of junctions that showed a lateral tip tilt (Figure 2a,b) was ≈−0.8 V, hence significantly greater than the ≈−0.3 to ≈−0.5 V of static silicon–platinum junctions (Figure 3a,b). Such negative shift is the result of a flexoelectric voltage term, which has the polarity of a reverse bias and results from the silicon strain gradient caused by the electrostatic‐driven tip rotation. Further mechanistic insights would be at this stage speculative as dynamic changes to the energy levels of a Schottky junction under a dynamic strain gradient remain poorly understood.^[^ ^60^ ^]^

**Figure 4:**
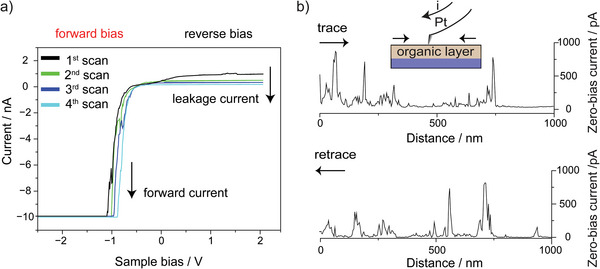
a) Consecutive color‐coded *I–V* curves acquired at a Pt AFM tip‐n‐type Si(211) **S**‐**1** junction under an applied normal load of 100 nN and at a sweep rate of 8 V s^−1^. b) Trace (left to right) and retrace (right to left) consecutive AFM scans acquired over the same sample location.

We then performed additional controls to further clarify both the delay between the flexoelectric effect and the tip‐tilt, as well as the potential involvement of galvanic (corrosion) processes. Consecutive *I–V* scans recorded over the same sample location (**Figure** **4**a) returned a progressive decay of the leakage current over successive scans along with an increase in forward current. This agrees with the flexoelectric term eventually disappearing as the movement subdues. It also suggests an unlikely role of corrosion, as the “oxidized” diode would not revert to a non‐leaky status as it does. Further, if silicon corrosion was involved, such corrosion current should decrease over time as silicon oxide grows. This is not what the data indicates. Sequential trace and retrace AFM scans in Figure 4b show that sample locations characterized by a significant tribocurrent retain this characteristic over time. We further ruled out galvanic corrosion by observing that the direction of the galvanic voltage does not depend, unlike the flexovoltage (Figure 1c,d), on the silicon doping type. Both n‐ and p‐type silicon samples immersed in an electrolytic solution, and then mechanically damaged to deliberately promote their corrosion, showed, as expected for a corroding substrate, a shift in open circuit potential towards more cathodic voltages, irrespective of the doping type (Figure S18, Supporting Information).

Finally, we tested our junctions when operated as a sliding TENG, with a representative zero‐bias current map (C‐AFM) shown in Figure S19 (Supporting Information). The tip slides along the S‐**1** surface generating a DC output that bears the sign of a reverse current, and with an average current density of ≈5.5 × 10^6^ A m^−2^. The performances of this TENG design are benchmarked in Table S1 (Supporting Information) against those of alternative TENG systems.

**Figure 5:**
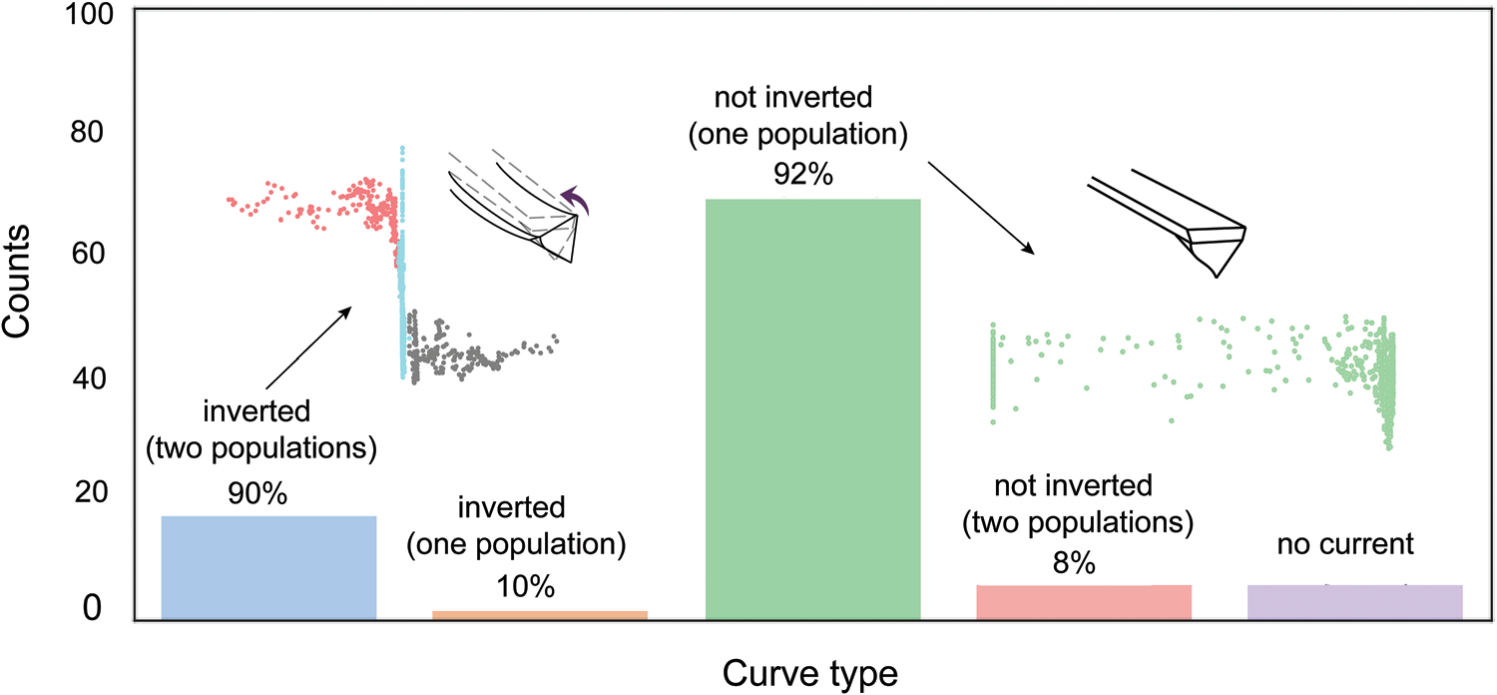
Analysis of 100 *I–V* curves recorded between a Pt AFM tip and n‐type Si(211) **S**‐**1** samples (applied normal load of 100 nN, voltage sweep rate of 8 V s^−1^).

We conclude with a quantitative analysis (**Figure** **5**) on the fraction of samples characterized by a simultaneous *I–V* inversion (RR below ≈0.3) and the presence of two discrete populations in the lateral tilt recorded during the *I–V*s. A remarkable 90% of the inverted *I–V* curves also showed two populations of lateral deflection. As discussed above, this mechanical tip instability causes a directional—additional reverse bias—parasitic flexovoltage. Furthermore, a compelling 92% of the samples with large RR (“not inverted” samples) were mechanically stable.

## 3 Conclusion

In this study we have revealed the presence of a directional “parasitic” reverse bias term in mechanically unstable Schottky junctions made between a platinum AFM tip and a monolayer‐modified oxide‐free silicon crystal. We have explained this additional reverse bias term as a silicon flexoelectric effect and assigned the origin of the mechanical instability to a semiconductor‐to‐metal electrostatic attraction, which surprisingly does not scale with the capacitance of the junction. The parasitic flexoelectric voltage adds to the external (instrumental) bias and leads to current fluctuations in current–voltage (*I*–*V*) measurements. The silicon strain gradient that leads to the flexoelectric disturbance can be detected as it is immediately preceded by a lateral deflection of the AFM cantilever. Such deflection is found in most “leaky” nanoscopic junctions, and we bring evidence that it is cause, not effect, of such a large leakage current.

We show the additional “flexoelectric” term is doping type dependent (i.e., its direction changes from n‐ to p‐type Si), it is linked to the electrostatic tip‐substrate attraction, and it is not a corrosion or heating effect. The work deepens our understanding of Schottky diodes, explains a parasitic effect in electrical AFM measurements and will aid the design and characterization of devices based on out‐of‐equilibrium diodes such as DC‐TENGs. We believe this work can aid in developing advanced sensing technologies and enhancing energy harvesting systems to fully leverage flexoelectric effects. Although large scale heterogeneous generators based on flexoelectricity are unlikely to be based on silicon, the reason being the availability of several materials with larger flexoelectric coefficients,^[^ ^66^ ^]^ the finding of this paper will be of value in nanoscale electrical measurements performed on silicon.

## 4 Experimental Section

### Chemicals and Materials

Redistilled dichloromethane (DCM), and Milli‐Q water (>18 MΩ cm resistivity) were used for substrate‐cleaning and surface‐modification procedures. Aqueous ammonium fluoride (40%, w/w), sulfuric acid (95–97%), and aqueous hydrogen peroxide (30% w/w) were of semiconductor grade and purchased from Honeywell. Methanol (≥99.8%), ammonium sulfite monohydrate (92%), aqueous hydrochloric acid (HCl, 35–37%), and 1,8‐nonadiyne (**1**, 98%) were purchased from Sigma–Aldrich. 2‐Propanol (>99.8%) was obtained from Ajax Finechem. Si(211) and Si(111) crystals, phosphorous‐doped (n‐type, 7−13 Ω cm, referred to as “lowly doped”) and boron‐doped (p‐type, 0.001 Ω cm, referred to as “highly doped”), prime grade, single‐side polished Si(211) crystals phosphorous‐doped (n‐type, 0.007−0.013 Ω cm, referred to as “highly doped”) were of prime grade, single‐side polished, with a mis‐cut angle of ± 0.05°, 500 µm in thickness and acquired from Siltronix, S.A.S (Archamps, France). Azidomethylferrocene (**2**) was prepared according to previously published procedures.^[^ ^38^ ^]^

### Silicon Surface Derivatization

Silicon wafers were cut into samples of either 10 × 10 mm (for AFM, current–voltage, and XPS measurements) 15 × 15 mm (electrochemical measurements), and then sequentially rinsed with DCM (3 × 50 mL), 2‐propanol (3 × 50 mL), and water (3 × 50 mL). Samples then underwent a 30‐min wash in a heated (100 °C) 3:1 mixture (v/v) of concentrated sulfuric acid (98%) and hydrogen peroxide (30%). Cautionary note: Piranha solution reacts violently with any organic material. Following the Piranha wash, samples were rinsed with water (3 × 50 mL) and then etched (with the polished side facing upwards) for 13 min in either aqueous 40% ammonium fluoride under ambient light (fluorescent tubes, ≈4.1 mW cm^−2^) for highly doped Si(111) and Si(211) samples, or under dark in a mixture of MeOH and aqueous 40% NH_4_F (1:10, v/v) for lowly doped Si(211) samples. The etching solutions had been previously deoxygenated by bubbling argon for 15 min (>99.99%, Coregas). Approximately 50 mg of ammonium sulfite was added to the etching bath as an oxygen scavenger. Hydrogen‐terminated samples (Si─H) were then rinsed once with water, twice with DCM (3 × 50 mL), and then blow‐dried using a stream of nitrogen gas. The Si─H surfaces were promptly subjected to a reaction with a small amount of compound **1** (Scheme 1), following slight adjustments to a previously reported hydrosilylation procedure. Briefly, the liquid diyne **1** stock was purged with argon for at least 15 min, and approximately 0.1 mL of it was applied to cover the Si─H surface. The sample rested for 2 h under nitrogen, positioned ≈20 cm from a 312 nm light source (Vilber, VL‐215.M, nominal power output of 30 W). A 1 mm thick quartz plate (part 01031‐AB, SPI Supplies) covered the liquid sample surface to limit its evaporation. The reaction was terminated by removing the acetylene‐terminated monolayer (**S**‐**1**) from the UV chamber, followed by extensive rinsing with DCM. **S**‐**1** samples were stored for 24 h under DCM at +4 °C before analysis.

**S**‐**1** samples were further derivatized through a CuAAC reaction to prepare a ferrocene‐functionalized silicon surface (**S**‐**2**). To this end, **S**‐**1** samples were rinsed with 2‐propanol and then placed in a reaction tube containing 4 mL of **2** in 2‐propanol (0.5 × 10^−3^ m), 1 mL of aqueous copper sulfate (20 mol % relative to **2**), and 1 mL of aqueous sodium ascorbate (100 mol% relative to **2**). The reaction tube was sealed, and the CuAAC reaction was conducted at room temperature with gentle agitation while shielded from ambient light. After 30 min, the ferrocene‐modified electrode (**S**‐**2**) was removed from the vial and thoroughly rinsed with 2‐propanol, water, HCl (0.5 m), and DCM. Finally, the samples were dried under a stream of nitrogen gas before analysis.

### Atomic Force Microscopy (AFM)

AFM measurements were conducted using a Park NX10 instrument from Park Systems Corp., Korea. The instrument has a variable‐enhanced conductive (VECA) probe holder for conductive AFM. The AFM tips were solid platinum tips (RMN‐25Pt300B and RMN‐12Pt300B, Rocky Mountain Nanotechnology, Holladay) with resonance frequency of 14 kHz and a spring constant of 18 N m^−1^ for the RMN‐25Pt300B tips and resonance frequency of 9 kHz and a spring constant of 0.8 N m^−1^ for the RMN‐12Pt300B tips. The tip radius was approximately 18.2 nm for both tips. All measurements were conducted at room temperature (22 ± 2 °C) in ambient air with a relative humidity of approximately 75% unless specified otherwise. Experiments conducted at higher temperatures (40 ± 2 °C) were conducted incorporating a Peltier plate (part number 174‐3113, RS components, Australia). Electrical contact was ensured between the silicon sample and the metal sample stage/holder bypassing the Peltier plate with copper tape. A positive sign current indicates a tip‐to‐silicon electron flow. Samples were mounted on the AFM stage using carbon tape after scratching the silicon's back with SiC emery paper and applying a small amount of gallium–indium eutectic (99.9% Alfa Aesar) on the abraded surface. Imaging parameters were set to a size of 5 × 5 µm, a resolution of 256 points per line, a scan rate of 1.0 Hz, and an applied normal force of 360 nN. Data analysis was performed using XEI processing software (Park Systems), and the reported average and maximum current outputs are based on a minimum of three independent measurements. The vertical palette color legend (scale bar) for the AFM height does not cover the full scale; the scale bar maximum was chosen to better visualize lateral changes in the height images. The residual output (noise level) is approximately around 120 fA. Force–distance (F–d) measurements were employed to estimate adhesion forces and the flexoelectric response of the samples. The maximum deflection was set to 2000 nN, the ramp size to 300 nm and the ramp speed to 300 nm s^−1^. The applied load on *I–V* measurements was 100 nN unless specified otherwise. To calculate the adhesion force, the curves were processed with OriginPro 9.0 (OriginLab Corp.)

### Scanning Electron Microscopy (SEM)

Samples were mounted onto the conductive copper tape and visualized using scanning electron microscopy (SEM). SEM images were captured on an FEI's Verios SEM using an Everhart–Thornley detector (ETD) with a voltage of 5 kV and a probe current of 100 pA.

### Macroscopic Current–Voltage Electrical Measurements

Current–voltage (*I*–*V*) measurements were performed inside a grounded and light‐proof Faraday cage using a source measure unit (SMU, B2902A, Keysight) and a FeGaInSn drop as the top contact. GaInSn alloy with a composition of 62% gallium, 22% indium, and 16% tin by weight was acquired from Thermo Fisher Scientific and was kept in conditions devoid of air exposure before its utilization. The process of creating magnetic GaInSn was adapted from the methodology described by Chen et al.^[^ ^67^ ^]^ In summary, 2 g of the GaInSn alloy were carefully blended with 200 mg of iron particles (approximately 0.5 µm in size, purity of 99.98%, sourced from Alfa Aesar) within a 150 ml Pyrex container. Following this, the resultant magnetic GaInSn composite was subjected to purification through five consecutive rinses with 20 mL of a 0.5 m hydrochloric acid solution, after which it was preserved away from light, and submerged in a 0.5 m HCl solution until needed. The drop of FeGaInSn was dispensed on the silicon sample using a threaded plunger syringe (Gilmont, model GS‐1200A, 2 mL capacity, 0.002 mL graduation intervals, 24 G). A steel needle with a blunt tip (approximately 0.49 mm radius) was brought into contact with the GaInSn drop and connected to the SMU. The positioning of the needle in contact with GaInSn was controlled by a three‐axis micromanipulator (three DTS25 linear translation stages, Thorlabs). For *I–V* measurements the junction voltage was cyclically ramped between −2.0 and 2.0 V in steps of 55 mV. The bias is applied from the sample to the GaInSn top contact, and the terminal was configured to be grounded internally (sensing type set to four wires).

### Electrochemical Measurements

Cyclic voltammetry and open‐circuit potentiometry experiments were performed with a CHI650D electrochemical workstation (CH Instruments) and a three‐electrode, single‐compartment poly(tetrafluoroethylene) cell. The working electrodes were monolayer‐modified **S**‐**2** surfaces (15 × 15 mm), a platinum coil served as the counter electrode, and Ag/AgCl (in a 3.0 m aqueous NaCl solution) was employed as the reference electrode. The geometric area of the working electrode was set to 0.28 cm^2^ using a circular rectilinear cross‐sectional Kalrez gasket. Electrical contact between the working electrode and a copper plate was established following the same procedure described for the conductive AFM experiments. The electrolyte was aqueous 1.0 m perchloric acid, and all measurements were conducted in air at room temperature (22 ± 2 °C) and under illumination (660 nm light‐emitting diode, nominal power output 940 mW, Thorlabs part M660L4) coupled to a collimator adapter (SM1P25‐A, Thorlabs). Illumination of the silicon electrode was through the electrolyte compartment, the collimator–sample distance was approximately 10 cm, and illuminance was measured using a meter from Amprobe (model IC‐LM‐200). The light intensity at the electrode surface was generally ≈0.12 mW cm^−2^. Surface coverages of ferrocene molecules on **S**‐**2** samples are reported in mol cm^−2^. Coverage values were calculated from the cyclic voltammetry derived Faradaic charge, taken as the average of the anodic and cathodic scans background‐subtracted integrated current. The 99% confidence limit of the mean coverage was calculated following literature procedures.^[^ ^68^ ^]^

### X‐Ray Photoelectron Spectroscopy (XPS)

XPS was employed to analyze the atomic composition and chemical state of elements in the monolayers. An AXIS Ultra DLD spectrometer (Kratos Analytical Inc., Manchester, UK) with monochromatic Al Kα radiation (1486.6 eV) and a hemispherical analyzer (165 mm radius) was operated in fixed analyzer transmission mode at 2 × 10^−8^ Torr. The analysis area was 300 × 700 µm, and the photoelectron take‐off angle was perpendicular to the sample. Survey spectra resulted from the accumulation of five scans using 160 eV pass energy over a 0–1100 eV range in 0.5 eV steps. High‐resolution spectra (10 scan accumulations) were collected at a pass energy of 20 eV, with step sizes of 0.05 eV for Si 2p (96–108 eV) and 0.1 eV for C 1s (278–295 eV). The pass energy was set to 20 eV, the step size to 0.05 eV, and the dwell time to 200 ms. The XPS data underwent processing using CasaXPS software. Spectra analysis involved background subtraction using the U2 Tougaard routine and Lorentzian‐Asymmetric (LA, 1.53243) line shape. All reported energies are binding energies (eV) and are corrected by applying a rigid shift to bring the Si 2p_3/2_ emission to 99.5 eV.

## Conflict of Interest

The authors declare no conflict of interest.

## Supporting information

Supporting Information

## Data Availability

The data that support the findings of this study are available from the corresponding author upon reasonable request.
