# Supplementary material for: Schottky Diode Leakage Current Fluctuations: Electrostatically Induced Flexoelectricity in Silicon
Source: Adv Sci (Weinh). 2024 Aug 9;11(38):2403524. doi: 10.1002/advs.202403524 (PMC11481228; doi:10.1002/advs.202403524)
Supplement: Supplementary file 1 — Supporting Information [file ADVS-11-2403524-s001.docx]

*Supporting Information*

Schottky Diode Leakage Current Fluctuations: Electrostatically Induced Flexoelectricity in Silicon

*Carlos Hurtado^a^, Melanie MacGregor^b^, Kai Chen^c^ and Simone Ciampi^a,^**

^a^School of Molecular and Life Sciences, Curtin University, Bentley, Western Australia 6102, Australia

^b^Flinders Institute for Nanoscale Science and Technology, Flinders University, Bedford Park, South Australia 5042, Australia

^c^School of Molecular Sciences, The University of Western Australia, Crawley, Western Australia 6009, Australia

*Email: [simone.ciampi@curtin.edu.au](mailto:simone.ciampi@curtin.edu.au)

| 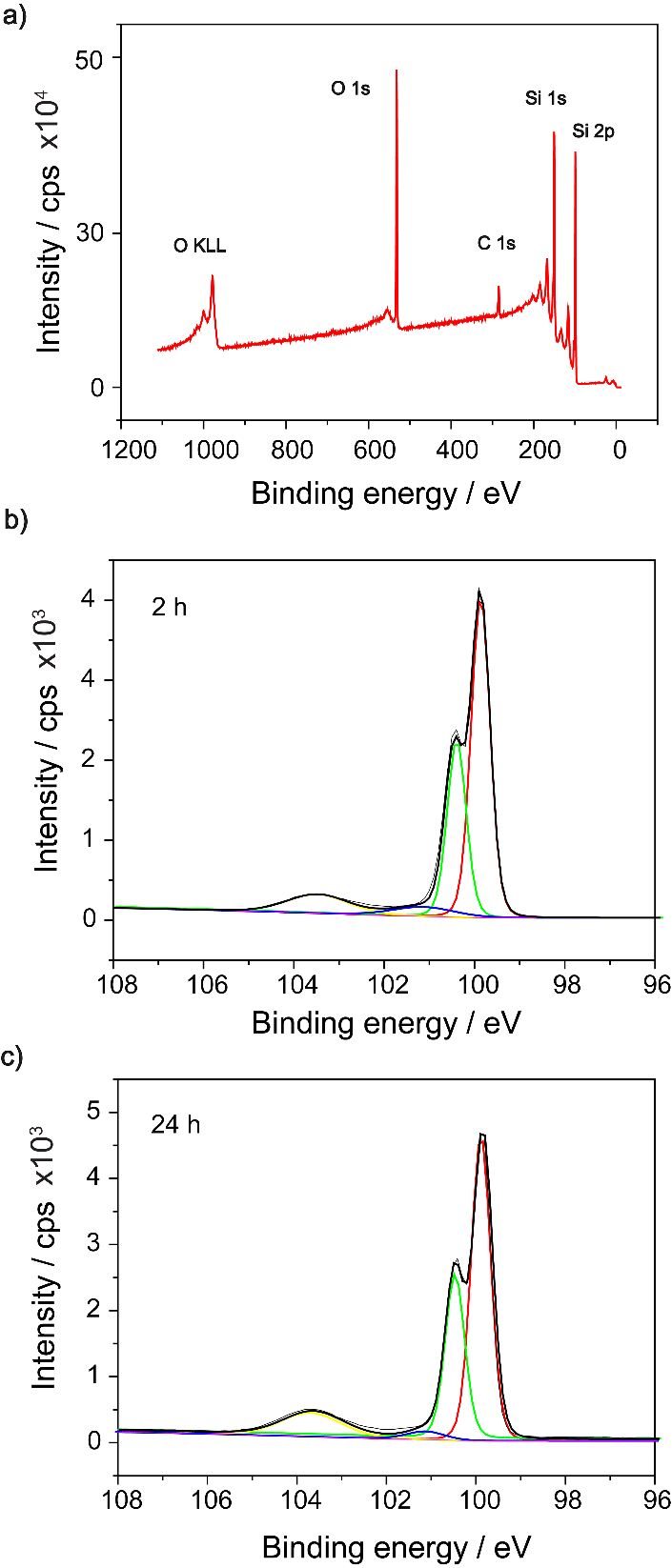 |
| --- |
| **Figure S1.** XPS data for Si–H samples made on n-type, lowly doped Si(211) crystals. The substrates were etched for 13 min in a mixture of MeOH and 40% NH_4_F (1:10) under dark. (a) XPS survey spectra. (b, c) Narrow scans of the Si 2p region conducted on the Si–H terminated samples exposed to ambient air for either (b) 2 h, or (c) 24 h. The Si 2p_3/2_ emission is at 99.5 eV. A noticeable high binding energy shoulder (0.40 eV from the Si 2p_1/2_ emission), is ascribed to Si−H species. Evident in the 102–105 eV region are photoelectrons originating from Si^(2)^, Si^(3)^, and Si^(4)^ oxides, which coalesce into a single band. |

| 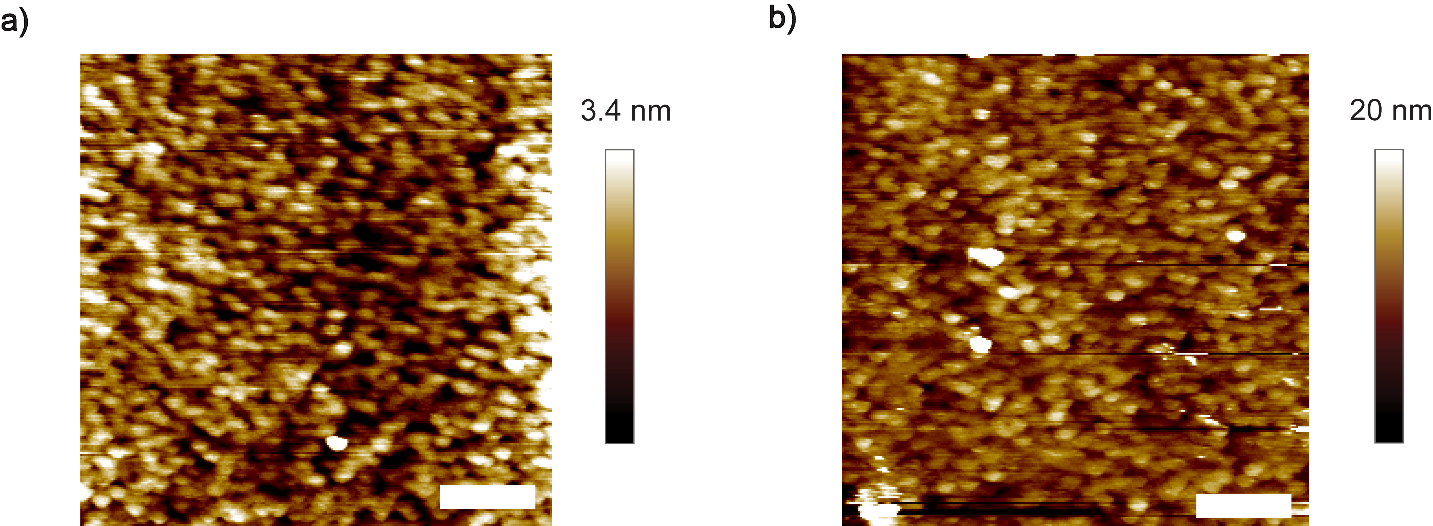 |
| --- |
| **Figure S2.** AFM topography images of Si–H terminated surfaces (n-type, lowly doped, Si(211) crystals) exposed to air for 2 h (a) and 24 h (b). Rounded topographical features (white dots) are attributed to oxidized surface sites. Scale bars in panels (a) and (b) are 1 μm. |

| 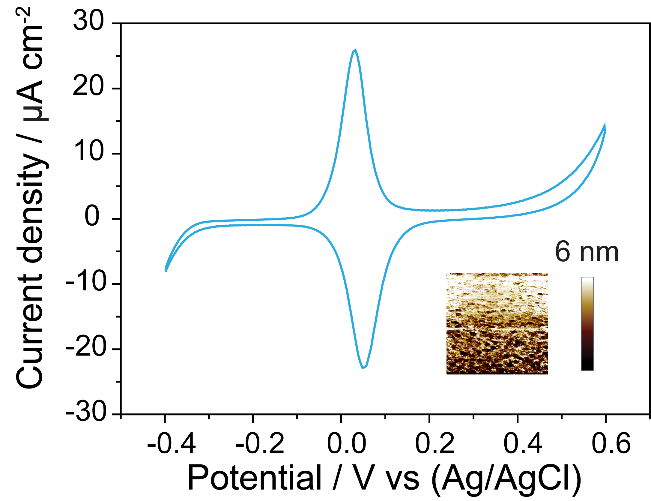 |
| --- |
| **Figure S3.** Representative cyclic voltammogram for a **S-2** sample made on a Si(211) and run as photoanode (n-type, lowly doped, 100 mV s^–1^, 1.0 M HClO_4_, red light illumination). The sample was etched in a mixture of MeOH and aqueous 40% NH_4_F (1:10, v/v) in the dark. The inset displays AFM topography image of the **S-1** sample (rms = ~3 nm). |

| 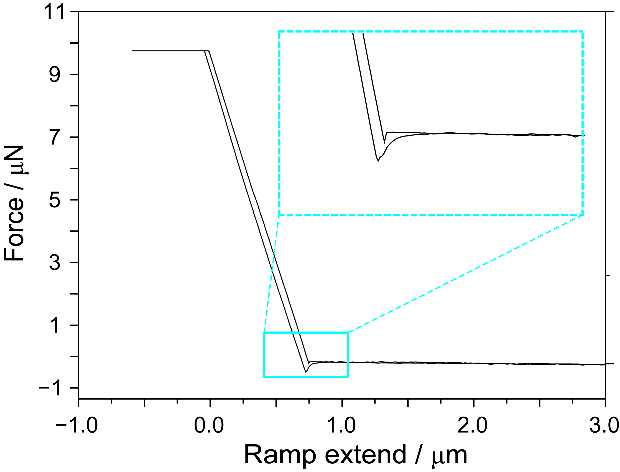 |
| --- |
| **Figure S4.** (a) A representative force–distance (F–d) AFM curve part of an adhesion force measurement. The inset shows the section of the curve used to estimate the adhesion force. |

| 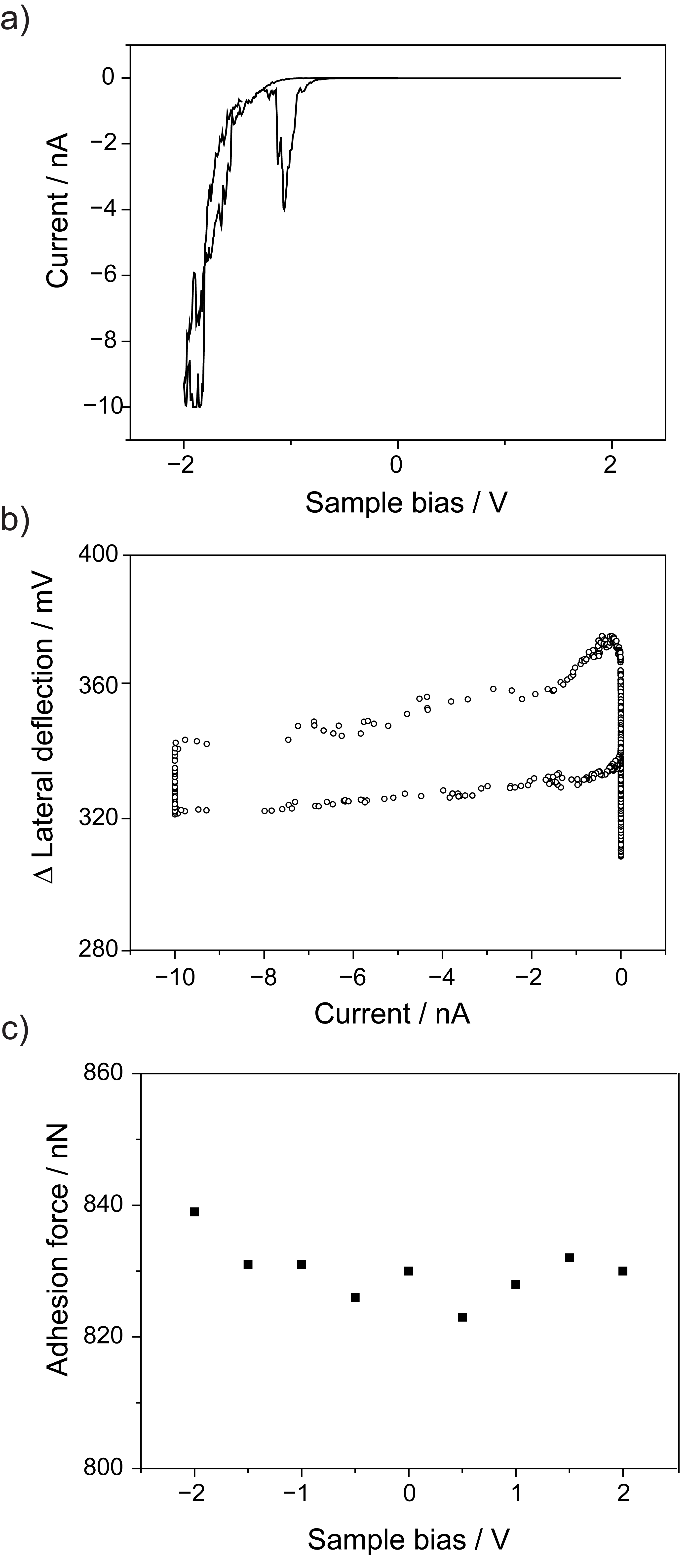 |
| --- |
| **Figure S5.** (a) Current–voltage (I–V) spectroscopy data acquired on a **S-1** sample grafted on highly doped Si(211). The force setpoint was of 100 nN, and the voltage sweep rate 8 V/s. The applied bias ranged from −2 V to +2 V. (b) Tip tilting–current plot corresponding to the experiment shown in (a), showing the lateral tip movement as function of the current crossing the Pt–silicon junction. The lateral deflection data suggest only a small tip deflection. (c) Tip–sample adhesion force as function of the externally applied bias (−2 V to +2 V). |

| 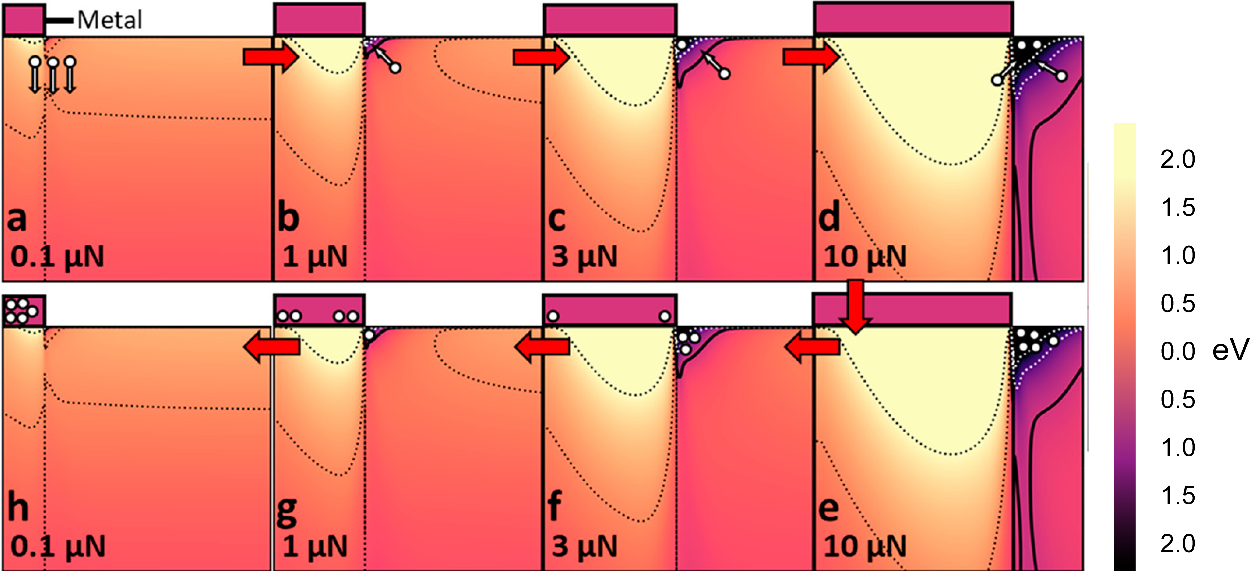 |
| --- |
| **Figure S6**. Proposed ratcheting mechanism for charge transfer in a PtIr–STO system. Reprinted (adapted) with permission from Nano Lett. 2022, 22, 10, 3914–3921. Copyright 2022 American Chemical Society. (a) At minimal applied force, the depletion potential is predominant. (b–d) As the force rises, electrons (illustrated as white circles with arrows) shift from areas with an increasing potential to areas with a decreasing potential. (e–g) Once the applied force diminishes, the quantity of available states within the potential well is reduced. This forces some electrons to move into the metal instead of crossing back over the barrier into the semiconductor (h). |

| 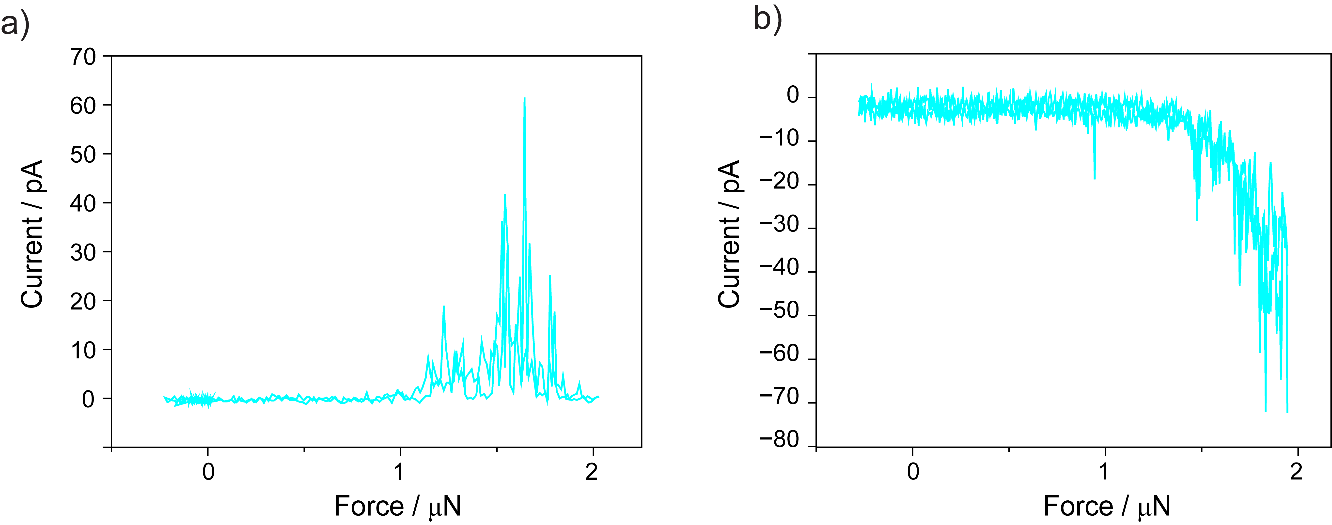 |
| --- |
| **Figure S7.** Current–force data acquired during AFM force–distance (F–d) measurements on Si(211) **S-1** monolayers prepared on either n-type (a), or p-type (b) Si(211) substrates. The F–d traces were acquired under zero external bias. The instrument routing is such that a current with a positive sign indicates a flow of electrons from the tip to the sample. |

| *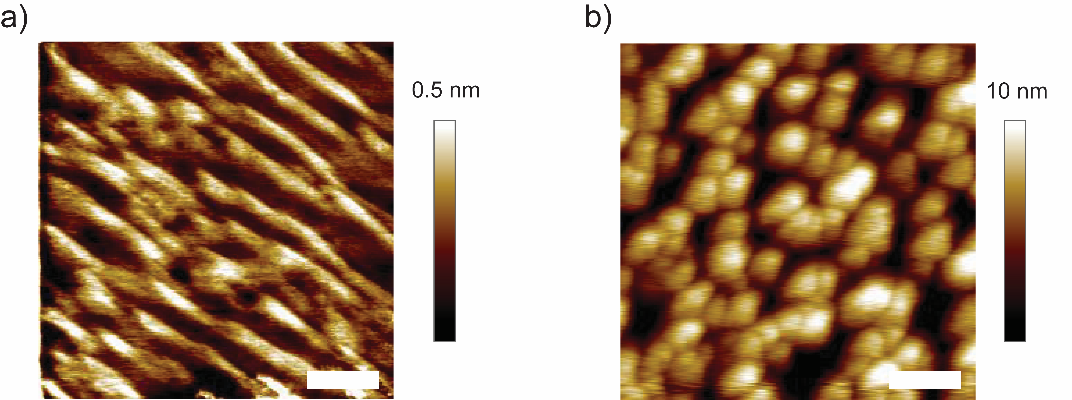* |
| --- |
| **Figure S8**. AFM topography images for **S-1** monolayers grafted on (a) Si(111) and on (b) Si(211) crystals, both n-type and lowly doped. The root-mean-square (rms) roughness of the Si(111) sample is ~ 0.3 nm. The rms value of the Si(211) sample is ~ 3 nm. The horizontal scale bars in panels (a) and (b) are 1 μm. |

| 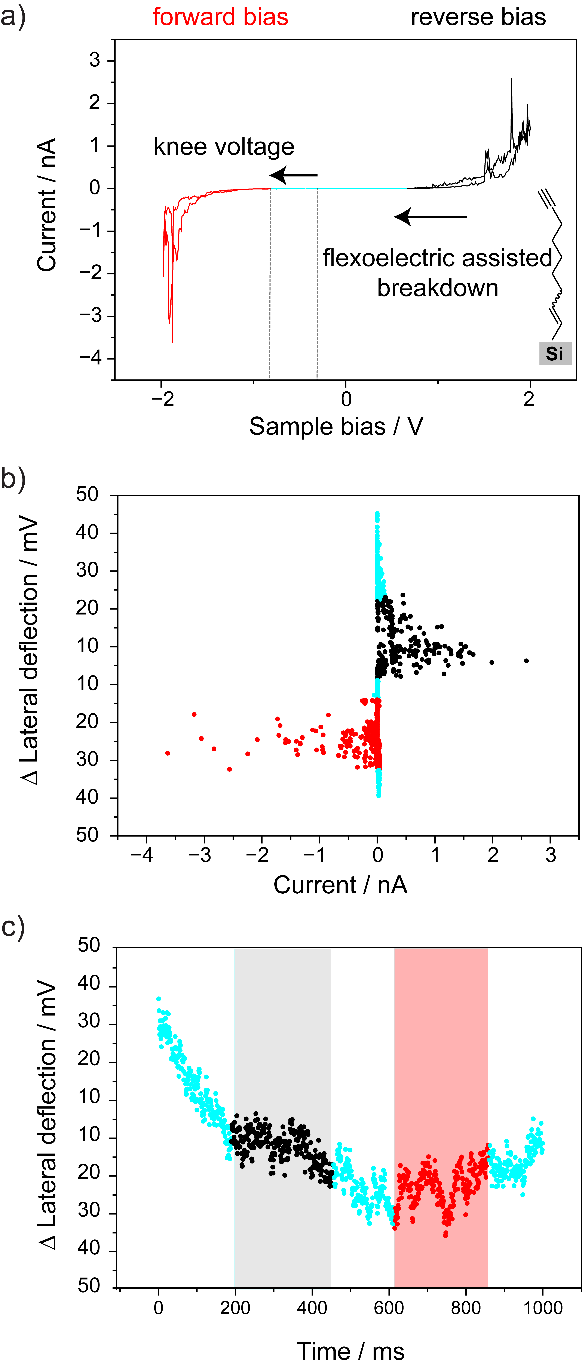 |
| --- |
| **Figure S9**. (a) A representative I–V curve acquired for a Pt AFM tip–n-type Si(111) **S-1** junction under an applied normal load of 100 nN, and at a voltage sweep rate of 8 V/s. The two vertical dashed lines indicate the conventional knee voltage for a Si/Pt junction (generally located between ~−0.3 and ~−0.5 V), and the “shifted” knee voltage (~−0.8 V) experimentally observed when a putative flexoelectric voltage term is present. Analogously, this flexoelectric term shifts the reverse breakdown voltage towards smaller applied reverse voltages. (b) Lateral deflection–current plot indicating a substantial tip-tilt during the I–V experiment shown in (a). (c) Plot of the lateral deflection data shown in (b) as a function of time during the voltage ramp. Shaded areas indicate regions with a less pronounced rate of deflection change (i.e. tilting less over time) corresponding to forward regions where knee voltage shifts negative (red) and reverse regions where the breakdown voltage shifts towards zero (black). |

| *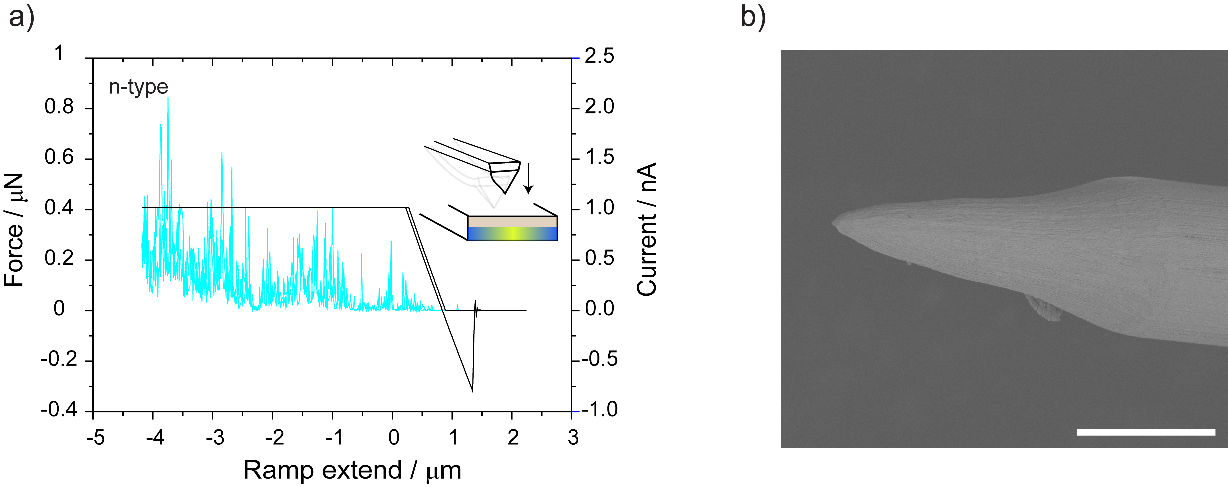* |
| --- |
| **Figure S10**. (a) High-speed capture current data acquired during AFM F–d measurements performed on **S-1** samples prepared on n-type Si(211). Experiments conducted with soft RMN-12Pt300B tips (spring constant of 0.8 N/m for the RMN-12Pt300B tips, versus 18 N/m of the RMN-25Pt300B). (b) SEM image of an RMN-12Pt300B tip. The scale bar is 10 μm. |

| 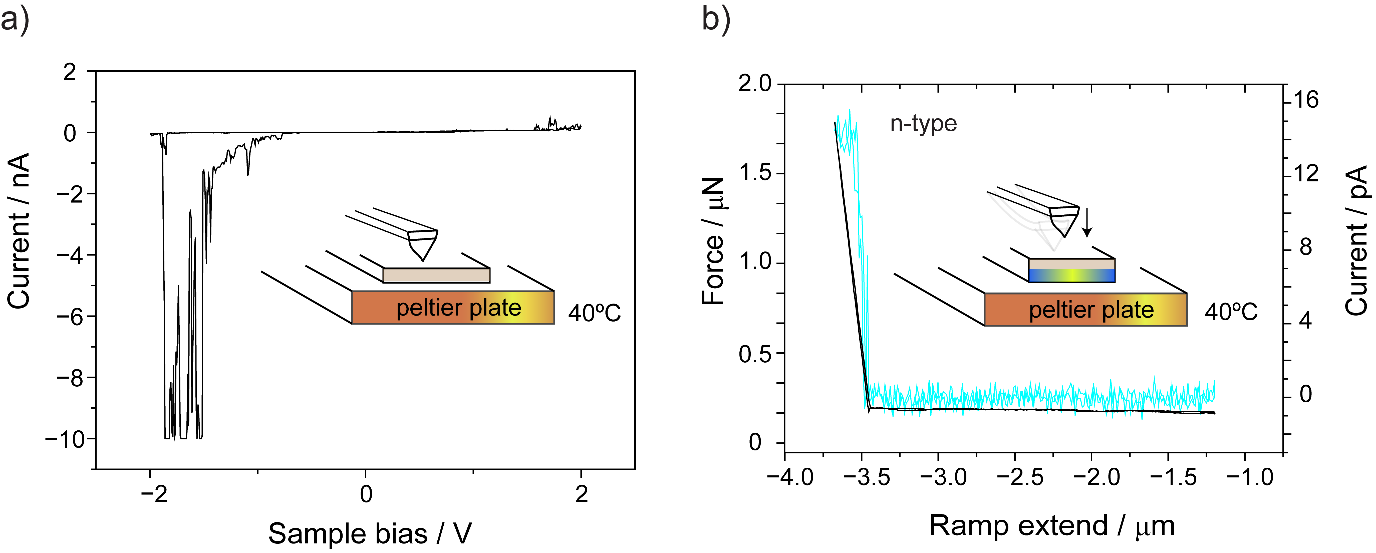 |
| --- |
| **Figure S11.** (a) Representative I–V curve acquired on Pt AFM tip–n-type Si(211) **S-1** junctions under an applied normal load of 100 nN at a voltage sweep rate of 8 V/s. (b) F–d measurements conducted on Si(211) **S-1** n-type. A Peltier plate schematically depicted in the inset, was used to increase the sample temperature to 40 ± 2 ºC. Electrical contact between the back of the silicon sample and the metal AFM sample stage/holder (not shown in figure) was realised by copper tape bypassing the Peltier plate. |

| 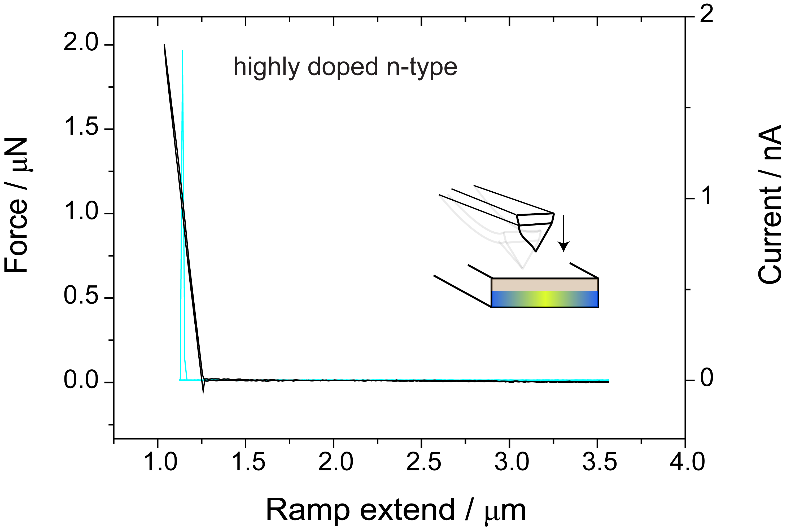 |
| --- |
| **Figure S12.** Representative AFM-based F–d measurement performed on a **S-1** sample prepared on highly doped Si(211) n-type. |

| 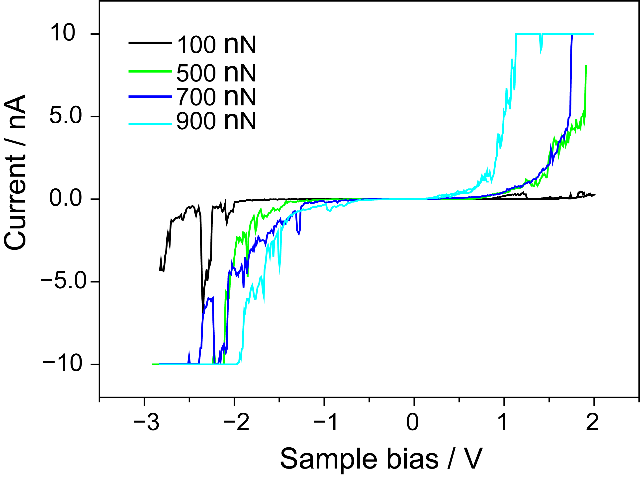 |
| --- |
| **Figure S13.** I–V measurements conducted on lowly doped Si(211) **S-1** n-type samples at a voltage sweep rate of 8 V/s. The normal load applied to the cantilever was varied as specified by the labels in figure. |

| 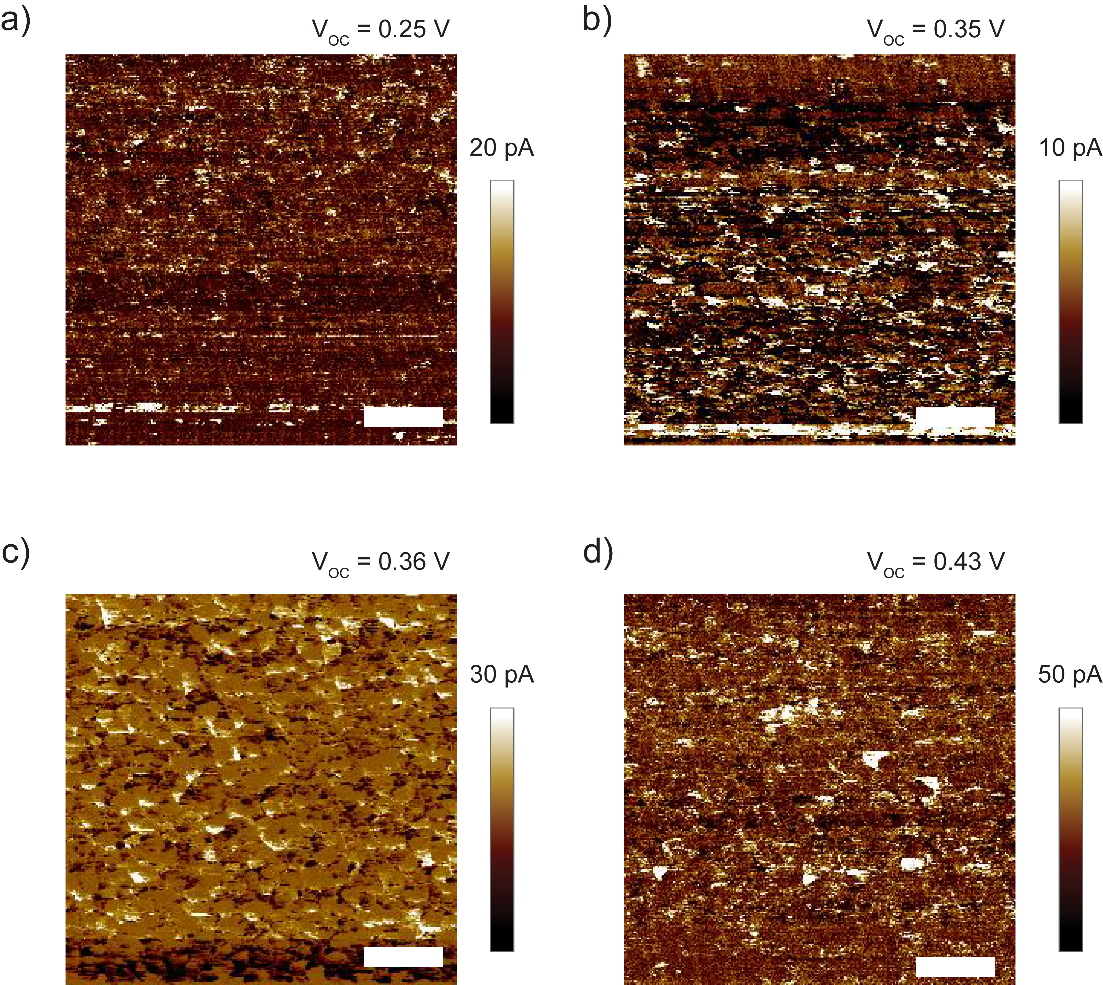 |
| --- |
| **Figure S14**. Experimentally determined open-circuit voltage (V*_OC_*) on Si(211) **S-1** surfaces. (a–d) C-AFM maps recorded on **S-1** samples with the platinum AFM tip moving along the surface at 1 Hz. The force setpoint was 100, 500, 700, and 900 nN for panels (a–d), respectively. The bias applied to the silicon sample corresponds to the voltage required to bring the current output to noise level at each force setpoint. |

| *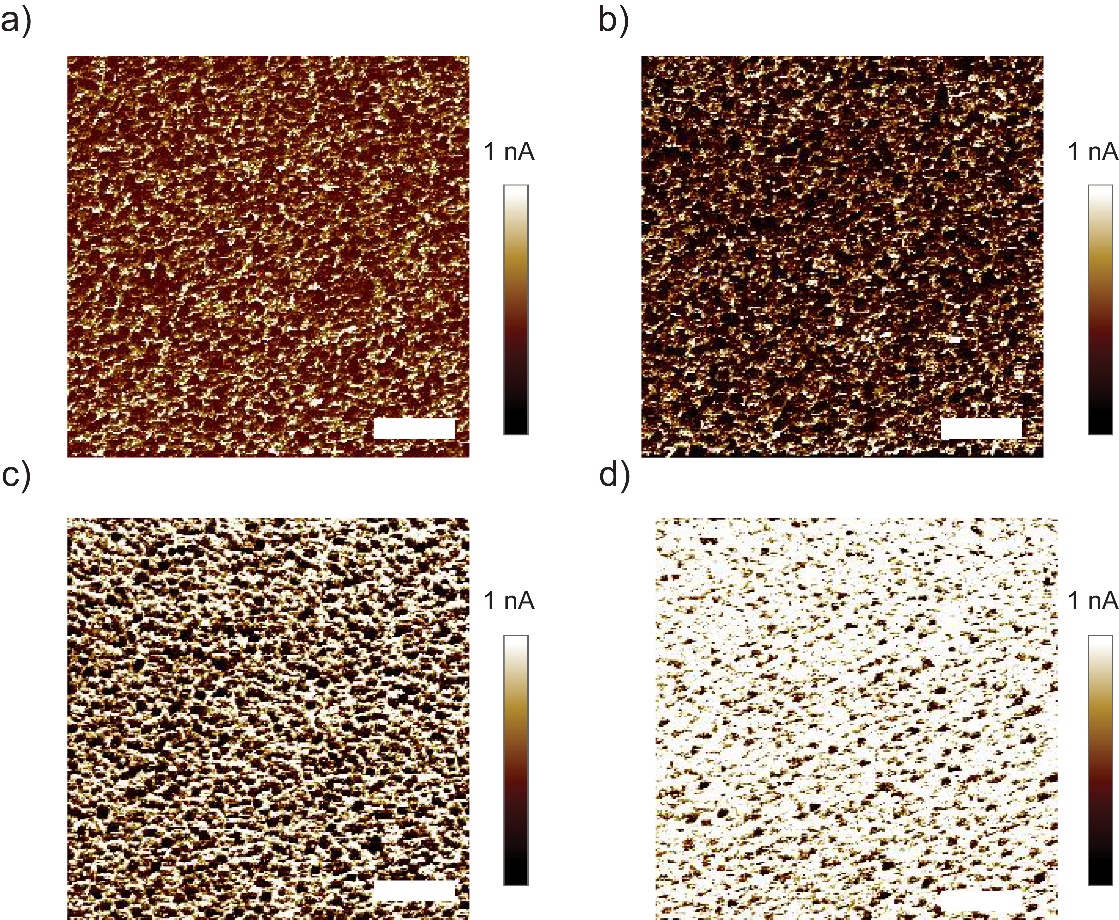* |
| --- |
| **Figure S15**. Experimentally determined short-circuit current (I*_SC_*) for Si(211) **S-1** surfaces. (a–d) Zero-bias C-AFM maps recorded on **S-1** samples with the platinum AFM tip moving along the surface at 1 Hz. The force setpoint was 100, 500, 700, and 900 nN, panels (a–d), respectively. |

| 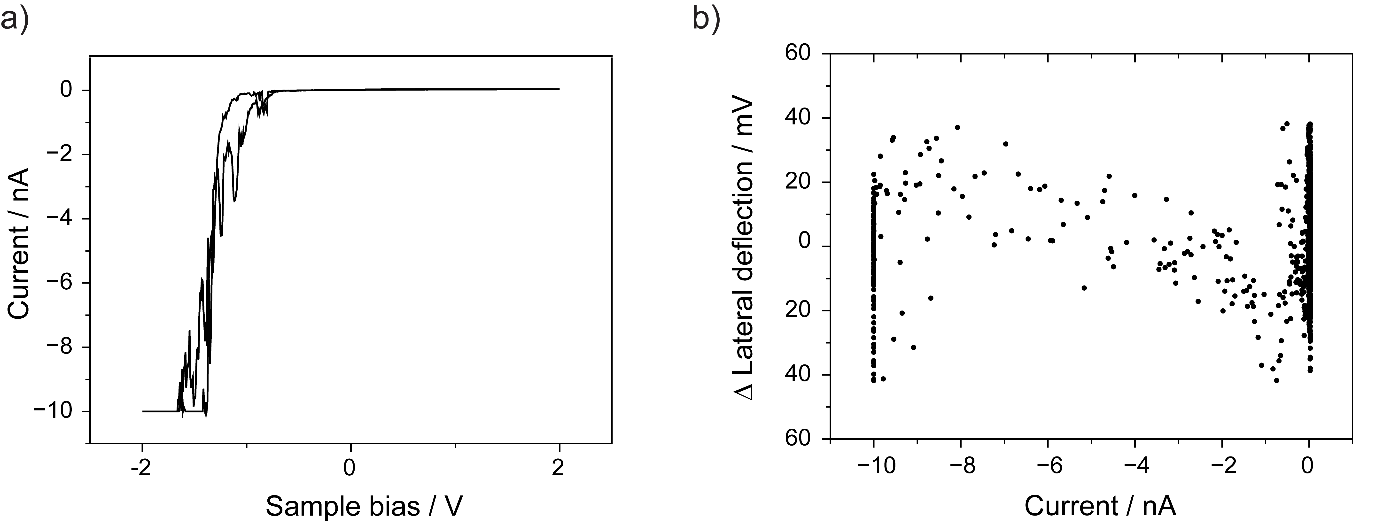 |
| --- |
| **Figure S16.** (a) Experimental current–voltage (I–V) spectroscopy data acquired on **S-1** lowly-doped n-type functionalized Si(211) surfaces with an applied load of 100 nN, at 0.01Hz (0.08 V/ s sweep rate) and an applied bias which ranged from −2 V to +2 V. (b) Tip tilting–current plot corresponding to the experiment in (a), tracking the lateral tip displacement as a function of the current crossing the junction. Lateral force values indicate low or negligible lateral tip displacement. |

| 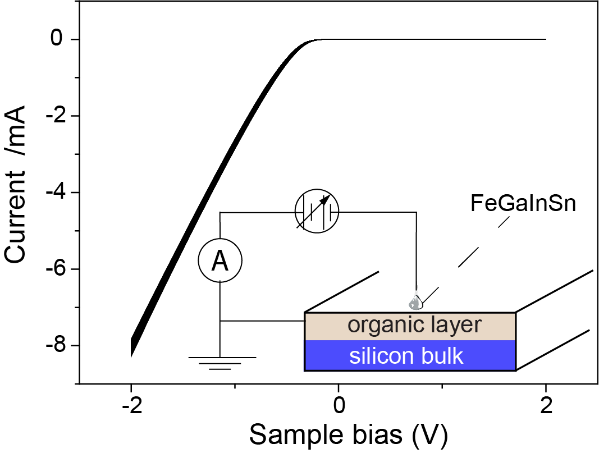 |
| --- |
| **Figure S17.** Evolution of current–voltage (I–V) curves over 50 continuous voltage cycles (∼1.5 h of sampling time, −2 V to +2 V) recorded on a **S-1** Si(211) surface with FeGaInSn top contact, in ambient air at a voltage sweep rate of 55 mV/s. The inset represents the scheme of the measurement circuit. |

| 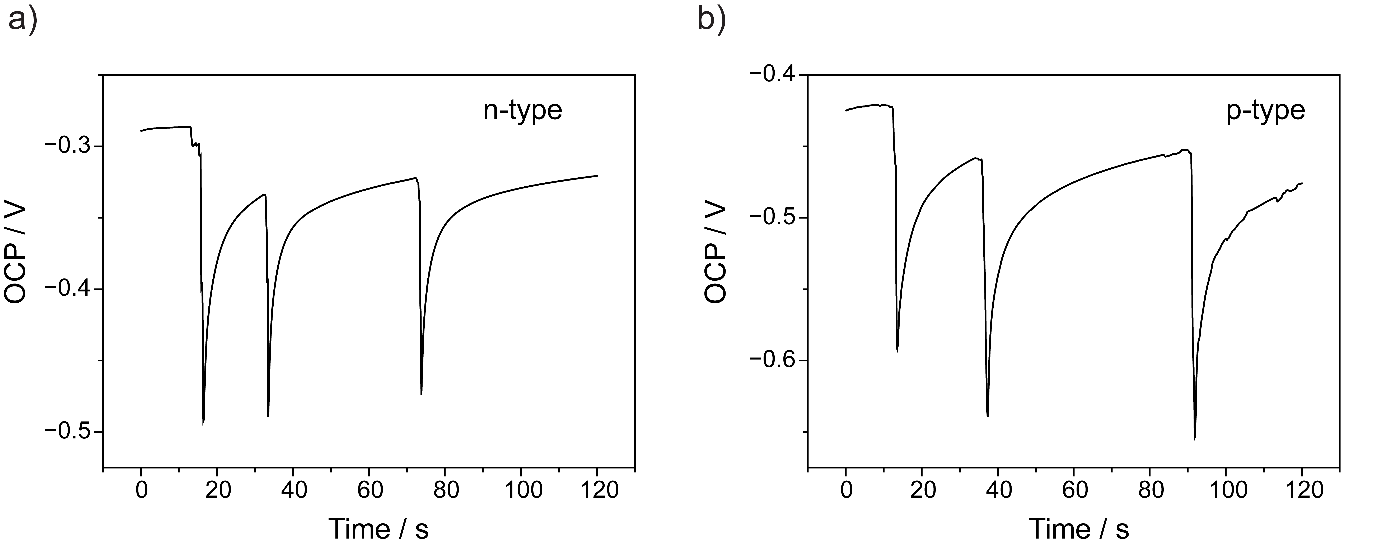 |
| --- |
| **Figure S18.** (a) Evolution of the open-circuit potential (OCP, working vs reference electrode) over time of a Si(211) lowly-dope n-type (a), and of a lowly-doped p-type (b) electrodes. In both panels, three cathodic OCP spikes indicate the build-up of an open-circuit electronic “pressure” following the in-situ mechanically induced (surface scribing with a diamond scribe) oxidation of the freshly exposed silicon surface. Samples are immersed in aqueous 1.0 M HClO_4_ electrolyte. |

| 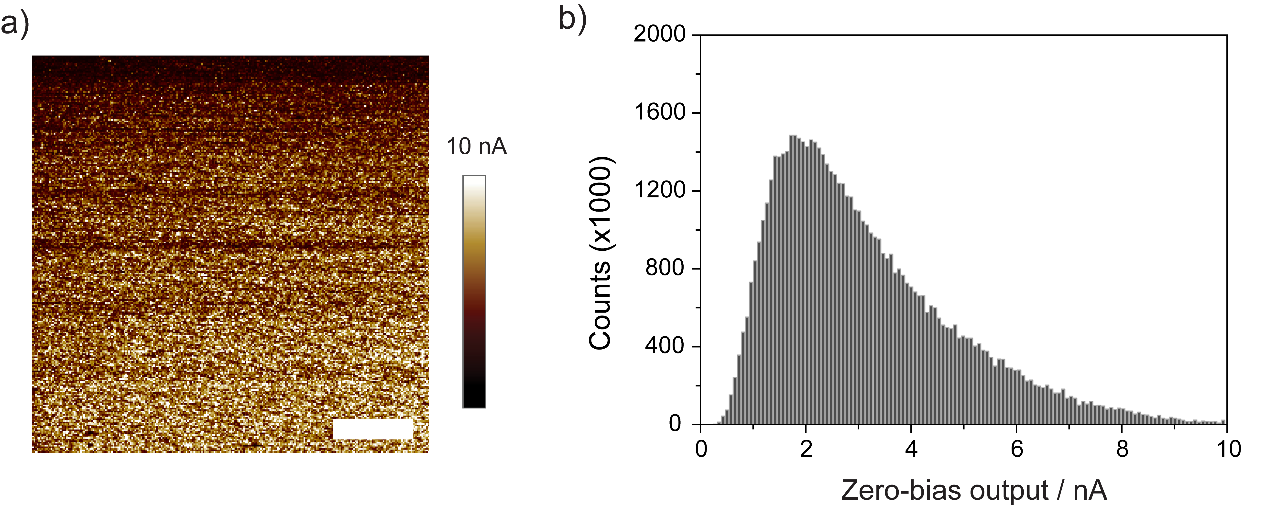 |
| --- |
| **Figure S19.** C-AFM map recorded on a **S-1** sample, at zero-voltage bias, and with the platinum AFM tip moving along the surface at 1 Hz (force setpoint of 360 nN). The horizontal scale bar in panel (a) represents 1 μm. (b) Histogram plot of the current output for the C-AFM map shown in panel (a). Counts indicate the number of pixels of a given current bin (x-axis), with a total of ∼65K pixels being sampled in a single C-AFM map. |

**Table S1. Performances of various TENGs designs.**

| **Material junction** | **Surface treatment** | **Operation mode** | **I*_sc_***  **(nA)** | **Load**  **(nN)** | **V*_oc_***  **(V)** | **J_avg._**  **(A/m^2^)** | **ϕ_B_**  **(eV)** | **Ideality**  **factor** | **Ref** |
| --- | --- | --- | --- | --- | --- | --- | --- | --- | --- |
| Si(111) and Si(100)/ [Pt] | SAMs functionalized  (1,8-nonadiyne) | Sliding |  |  |  | 2.9 × 10^5^ | 0.3 |  | [1] |
| Si(111)/ [Pt] | SAMs & CuAAC  (–NH_2_, –OH, –CH_3_) | Sliding |  |  |  | 4.6 × 10^6^ |  |  | [2] |
| Si(211)/ [Pt] | SAMs functionalized  (–CH_3_, –OH, –C≡CH) | Sliding |  |  |  | 4.9 × 10^9^ |  |  | [3] |
| Si(211)/ [Pt] | SAMs functionalized  (1,8-nonadiyne) | Static |  | 100 |  |  |  | 6.8 | This work^e^ |
| Si(211)/ [Pt] | SAMs functionalized  (1,8-nonadiyne) | Static |  | 500 |  |  |  | 8.2 | This work^e^ |
| Si(211)/ [Pt] | SAMs functionalized  (1,8-nonadiyne) | Static |  | 700 |  |  |  | 12.5 | This work^e^ |
| Si(211)/ [Pt] | SAMs functionalized  (1,8-nonadiyne) | Static |  | 900 |  |  |  | 19.0 | This work^e^ |
| Si(211)/ [Pt] | SAMs functionalized  (1,8-nonadiyne) | Sliding |  | 100 | 0.25 | – | – | – | This work^b^ |
| Si(211)/ [Pt] | SAMs functionalized  (1,8-nonadiyne) | Sliding |  | 500 | 0.35 | – | – | – | This work^b^ |
| Si(211)/ [Pt] | SAMs functionalized  (1,8-nonadiyne) | Sliding |  | 700 | 0.37 | – | – | – | This work^b^ |
| Si(211)/ [Pt] | SAMs functionalized  (1,8-nonadiyne) | Sliding |  | 900 | 0.42 | – | – | – | This work^b^ |
| Si(211)/ [Pt] | SAMs functionalized  (1,8-nonadiyne) | Sliding | 0.2^a^ | 100 | – | 4 × 10^6^ | 0.24 |  | This work ^c, d^ |
| Si(211)/ [Pt] | SAMs functionalized  (1,8-nonadiyne) | Sliding | 0.6^a^ | 500 | – | 5 × 10^6^ | 0.22 |  | This work ^c, d^ |
| Si(211)/ [Pt] | SAMs functionalized  (1,8-nonadiyne) | Sliding | 0.8^a^ | 700 | – | 6 × 10^6^ | 0.20 |  | This work ^c, d^ |
| Si(211)/ [Pt] | SAMs functionalized  (1,8-nonadiyne) | Sliding | 1.0^a^ | 900 | – | 7 × 10^6^ | 0.19 |  | This work ^c, d^ |
| PTFE/ [Cu] | Pre-charged PTFE | Sliding | 200 |  | 50 |  |  |  | [4] |
| Si/ [graphene] | HF etched | Sliding |  |  |  | 40.0 |  |  | [5] |
| Si(100)/ [graphite] | Buffered oxide etched | Sliding |  |  |  | 210 |  |  | [6] |
| p-type Si/ [n-type Si] | HF etched | Sliding | 50 |  |  |  |  |  | [7] |
| Si/ [black phosphorus] | Deposition  (AlN, HfO_2_, Al_2_O_3_) | Sliding |  |  | 6.1 | 124 |  |  | [8] |
| n-type Si/ [n-type Si] | HF etched | Sliding |  |  | 0.35 | 214 |  |  | [9] |
| p-type Si(111)/  [diamond coated Si tip] | Molecular beam epitaxy  (InP) | Sliding |  |  | 15× 10^−3^ | 2 × 10^4^ |  |  | [10] |

1. I_SC_, short-circuit current. Represents the average current at zero bias (under sliding at different set point).
2. V_OC_, open-circuit voltage. Represents the nullifying voltage (under sliding at different force set point) to bring the leakage current to noise level.
3. J_avg,._ Average current density calculated by estimating the tip–sample contact area using the Derjaguin–Muller–Toporov (DMT) model.
4. ϕ_B_, Schottky barrier height calculated by applying the thermionic emission equation, using a the average current density (C-AFM) calculated at different setpoint forces.
5. Ideality factor calculated from the slope of the linear region of the natural logarithm of the forward current density versus voltage plot.

[1] S. Ferrie, N. Darwish, J. J. Gooding, S. Ciampi, *Nano Energy* **2020**, *78*, 105210.

[2] S. Ferrie, A. P. Le Brun, G. Krishnan, G. G. Andersson, N. Darwish, S. Ciampi, *Nano Energy* **2022**, *93*, 106861.

[3] X. Lyu, S. Ferrie, A. Pivrikas, M. MacGregor, S. Ciampi, *Nano Energy* **2022**, 107658.

[4] Z. You, S. Wang, Z. Li, Y. Zou, T. Lu, F. Wang, B. Hu, X. Wang, L. Li, W. Fang, Y. Liu, *Nano Energy* **2022**, *91*, 106667.

[5] S. Chen, D. Liu, L. Zhou, S. Li, Z. Zhao, S. Cui, Y. Gao, Y. Li, Z. L. Wang, J. Wang, *Adv. Mater. Technol.* **2021**, *6*, 2100195.

[6] S. Lin, Y. Lu, S. Feng, Z. Hao, Y. Yan, *Adv. Mater.* **2018**, *31*, 1804398.

[7] X. Huang, X. Xiang, J. Nie, D. Peng, F. Yang, Z. Wu, H. Jiang, Z. Xu, Q. Zheng, *Nat. Commun.* **2021**, *12*, 2268.

[8] R. Xu, Q. Zhang, J. Y. Wang, D. Liu, J. Wang, Z. L. Wang, *Nano Energy* **2019**, *66*, 104185.

[9] Y. Lu, S. Feng, R. Shen, Y. Xu, Z. Hao, Y. Yan, H. Zheng, X. Yu, Q. Gao, P. Zhang, S. Lin, *Research* **2019**, *2019*, 5832382.

[10] Y. Lu, Q. Gao, X. Yu, H. Zheng, R. Shen, Z. Hao, Y. Yan, P. Zhang, Y. Wen, G. Yang, S. Lin, *Research* **2020**, *2020*, 5714754.

[11] V. A. Sharov, P. A. Alekseev, B. R. Borodin, M. S. Dunaevskiy, R. R. Reznik, G. E. Cirlin, *ACS Appl. Energy Mater.* **2019**, *2*, 4395.

**References**
